# CAMTA1-immunonegative epithelioid hemangioendotheliomas of the liver: a clinicopathological and molecular analysis of seven cases

**DOI:** 10.3389/fonc.2025.1478036

**Published:** 2025-02-18

**Authors:** Yang Nie, Wenyi Jing, Xuanxuan Zheng, Xin He, Min Chen, Hongying Zhang

**Affiliations:** Department of Pathology, West China Hospital, Sichuan University, Chengdu, China

**Keywords:** epithelioid hemangioendothelioma, liver tumor, WWTR1::CAMTA1 fusion gene, YAP1::TFE3 fusion gene, molecular analysis

## Abstract

**Background:**

Epithelioid hemangioendothelioma (EHE) is a rare malignant vascular tumor. Most EHEs (>90%) cases harbor *WWTR1::CAMTA1* fusion gene, and CAMTA1 immunohistochemistry (IHC) is a highly sensitive and specific tool for EHE diagnosis. However, there exist CAMTA1-immunonegative cases, the majority of which harbor *YAP1::TFE3* fusion, with a few cases having more rare fusions. Liver is one of the most common sites of EHE, where the *CAMTA1* subtype dominates, and the other variants are extremely rare. Hence, we focused on the hepatic CAMTA1-immunonegative EHEs to analyze the clinicopathological and molecular features of these peculiar cases.

**Methods:**

The SNOMED search of the hospital pathology files between January 2016 to November 2023 identified 57 hepatic EHEs and 7 cases were CAMTA1-immunonegative. Fluorescence *in situ* hybridization (FISH), next generation sequencing (NGS) and Sanger sequencing were performed to identify the genetic change of the 7 cases.

**Results:**

This series included 3 females and 4 males, aged from 33 to 64 years. All the 7 cases were negative for CAMTA1 IHC. Four cases were positive for TFE3 IHC and exhibited *YAP1::TFE3* fusion. Another 3 cases were also negative for TFE3, while *WWTR1::CAMTA1* fusion were detected by NGS in 1 case and demonstrated by FISH in all the 3 cases. Morphologically, among the 4 *TFE3* rearrangement cases, 3 cases showed the *TFE3*-sutype morphologic appearance, while the histology of 1 case was similar to that of *CAMTA1-* subtype. In the 3 *CAMTA1*-rearranged lesions, 2 cases had classic EHE morphology, and 1 case exhibited atypical histology, with higher atypia and well-formed vessels. Surgical resection was performed on five cases and two cases were biopsied and received chemotherapy. Follow-up information was available in 6 patients (median 46 months), including 4 patients were alive without disease and 2 patients were alive with disease.

**Conclusion:**

Our study reported 7 CAMTA1-immunonegative hepatic EHEs and most of them were *TFE3*-rearranged EHEs with morphology variation. Moreover, there does exist the CAMTA1-immunonegative but *CAMTA1*-rearranged EHE cases. Therefore, the diagnosis of EHE should be based on morphology, combined with CAMTA1 and TFE3 IHC, and if necessary, supplemented by genetic analysis including FISH and NGS, to establish correct diagnosis.

## Introduction

Epithelioid hemangioendothelioma (EHE) is a rare malignant vascular tumor, whose clinical behavior is between hemangioma and malignant angiosarcoma (1). EHE affects all ages with a peak incidence in the 4-5th decades of life and a slight female preponderance in visceral tumors (2). Histologically, conventional EHE is composed of strands and nests of epithelioid cells in a myxohyaline stroma, with the presence of intracytoplasmic vacuoles which may contain erythrocytes. In 2001, Mendlick et al. found recurrent chromosomal translocation, involving 1p36.3 and 3q25 in EHE, and then Errani et al. demonstrated this recurrent translocation result in the *WWTR1::CAMTA1* fusion gene (3, 4). Hence, CAMTA1 immunohistochemistry (IHC) has been developed as a sensitive and specific tool for the diagnosis of EHE cases, and for the challenging cases, fluorescence *in-situ* hybridization (FISH) for the identification of *WWTR1::CAMTA1* fusion could further confirm the diagnosis (5, 6).

While a subset of EHE were found to be CAMTA1-immunonegative and fusion-negative, with distinctive morphology, such as abundant eosinophilic cytoplasm, and well-defined vascular channels. These cases were proved to be *TFE3*-rearranged, harboring *YAP1::TFE3* fusion, and TFE3 IHC serve as a useful screening tool for this subtype (7). Although most CAMTA1-negative cases are proved to be *TFE3*-subtype. In our clinical practice, we found there existed CAMTA1-immunonegative, but *CAMTA1*-rearranged EHE cases, and such peculiar cases were also reported in previous studies, with *CAMTA1*-rearrangemnt or other exceedingly rare fusion variant, including *WWTR1*::*ACTL6A* and *WWTR1*::*TFE3* fusions (8–11).

Liver is one of the most common sites of EHE, in which the *CAMTA1*-subtype dominates (>90%), and the *TFE3*-subtype is extremely rare. Hence, we performed this study focusing CAMTA1-immunonegative EHEs involving the liver, to further elucidate the clinicopathological and molecular features of CAMTA1-immunonegative EHE lesions at our institution. To the best of our knowledge, this is the first study of these rare, peculiar hepatic CAMTA1-immunonegative EHE cases.

## Materials and methods

### Case selection

This study was approved by the Institutional Review Board of West China Hospital. A SNOMED search of hospital surgical pathology documents from January 2016 to November 2023 identified 145 EHEs, including 57 (39.3%) hepatic EHEs. Seven EHE cases with negative CAMTA1 expression were finally identified. All cases were independently reviewed by two pathologists with soft tissue tumor pathology expertise (H.Z. and X.H.). Clinical and follow-up information was collected from the clinical records and pathology reports.

### Immunohistochemistry

Immunohistochemical staining was performed on 4-μm-thick formalin-fixed, paraffin-embedded (FFPE) tissue sections using the EnVision Plus detection system (Dako, Carpinteria, CA). IHC were performed using the following antibodies: CAMTA1 (clone NBP1-93620, 1:200; Novus Biologicals, Littleton, CO, USA), TFE3 (clone MRQ-37, 1:50; Cell Marque, CA, USA), CD31 (clone JC70A, 1:200; Dako, CA,USA), CD34 (clone QBEnd10, 1:200; Dako, CA,USA), ERG (clone UMAB78, 1:200; ZSGB-Bio, Beijing, China), cytokeratin (clone AE1/AE3, 1:100; Dako, CA,USA), Epithelial Membrane Antigen (EMA, clone GP1.4, 1:150; ZSGB-Bio, Beijing, China), Ki-67 (clone MIB-1, 1:100; Dako, CA, USA). In the immunohistochemical evaluation of CAMTA1 or TFE3, cases showing significant nuclear staining in >5% of tumor cells were considered positive (8). The staining intensity of each case was divided into weak, moderate, or strong, and the extent of nuclear immunoreactivity was scored according to the percentage of positive tumor cells: 1+ (5% to 35%), 2+ (36% to 65%), and 3+ (66% to 100%) (5).

### Fluorescence *in situ* hybridization

FISH analysis was performed on seven tumors with available material, using the GSP *WWTR1*:: *CAMTA1* fusion gene probe (Anbiping, Guangzhou, China) and GSP *TFE3* break apart probe (Anbiping, Guangzhou, China) for the detection of *WWTR1::CAMTA1* and *TFE3* rearrangement, respectively. The FISH assays were performed on 4-μm-thick sections according to an established laboratory protocol (12). At least 100 nuclei were counted in each case and the tumor was interpreted as positive for *WWTR1*:: *CAMTA1* fusion when at least 10 out of 100 (10%) tumor cells showed a (yellow) fusion signal. The case was interpreted as *TFE3*-rearranged when at least 10 out of 100 (10%) tumor cells exhibited a split signal pattern which showed that the distance between the green and red signals was greater than the diameter of two signals.

### Reverse transcription polymerase chain reaction

Four cases with available material were analyzed by RT-PCR. Total RNA was isolated from 4-μm sections of FFPE tissue material using the High Pure FFPE RNA Micro Kit (Qiagen, CA, USA) according to the manufacturer’s instructions. CDNA was synthesized using the PrimeScript RT reagent kit (Takara, Tokyo, Japan). The PCR was performed according to standard procedures using the primers (*YAP1*-exon1-forward: 5’-CTCCGGAAGCTGCCCGACTCC-3’, and *TFE3*-exon4-reverse: 5’-ACAGGTACTGTTTCACCTGCT-3’). The PCR products were sequenced by the Sanger sequencing (Tsingke Biological Technology, Chengdu, China).

### Next-generation sequencing

Genomic DNA of case 3 was extracted from the formalin-fixed paraffin- embedded (FFEP) tissue using a QIAamp DNA Mini Kit (Qiagen, CA, USA) and then quantified by Nanodrop spectrophotometer (Thermo Fisher Scientific, DE, USA). Subsequently, the DNA was sheared, purified, ligated with adapters and used for the library construction, and then hybridized to a panel of 1021 genes containing whole exons of 312 genes, selected introns of 38 genes, and selected regions of 709 genes. Sequencing was performed with the Illumina gene^+^Seq 2000 system (Illumina, San Diego, CA, USA). DNA and RNA of case 7 were extracted from FFPE samples using a QIAamp DNA Mini Kit (Qiagen, Valencia, CA, USA) and RNeasy FFPE Kit (Qiagen, Valencia, CA, USA). The NGS were performed using a cancer-related DNA+RNA panel (YuansuS, OrigiMed). DNA and RNA library construction were performed according to the manufacturers’ instructions. The genes were captured and sequenced with a mean coverage of 700× on the Illumina gene+Seq 2000 system (Illumina, San Diego, CA, USA).

## Results

### Clinical characteristics

The clinical features of hepatic EHE are summarized in **Table 1**. This series included 4 males and 3 females (ratio 1.33:1), aged from 33 to 64 years (median 44 years; mean 47.8 years). The tumor size ranged from 1.6 to 9 cm (median was 2.7 cm; mean 3.6 cm). Four patients had clinical symptoms, including abdominal pain, cough, fever, and jaundice. The other 3 patients were asymptomatic, and the tumors were found incidentally. Six tumors occurred within the liver, and in 1 case the tumors involved the liver and lung simultaneously. Among the 6 cases, three patients had multicentric lesions involving the whole liver, and another three patients had solitary lesions, with 2 tumors occurring in the right lobe and 1 in the left lobe of the liver.

**Table 1:** Clinicopathological and molecular characteristics of 7 CAMTA1-immunonegative EHE cases.

Case (no.)	Age/Sex	Location	Symptom	Size (cm)	Morphology	Immunohistochemistry			Molecular results	Treatment	Outcome
						CAMTA1	TFE3	Other markers			
1	36/M	multiple intrahepatic lesions	fever, jaundice	2.5	large epithelioid cells in a solid growth pattern with pseudoalveolar architecture	–	+	CD31+,CD34+,PCK-,EMA-,Ki-67-15%	*TFE3-*rearranged(FISH); *YAP1::TFE3*(PCR)	SR	NA
2	62/M	left liver	mild jaundice	3.9	large epithelioid cells within inflammatory background and pseudoalveolar pattern	–	+	CD31+,CD34+,PCK-,EMA-,Ki-67-5%	*TFE3-*rearranged(FISH); *YAP1::TFE3*(PCR)	SR	NED/86mo
3	58/F	right liver	asymptomatic	1.6	medium-sized epithelioid cells, vascular channels containing erythrocytes	–	+	CD31+,CD34+,PCK-,EMA-,Ki-67-5%	*TFE3-*rearranged(FISH); *YAP1::TFE3*(PCR&NGS)	SR	NED/16mo
4	64/F	right liver /lung	cough	2.7	spindle cells in the myxoid stroma, intracytoplasmic vacuoles and signet-ring lumen	–	+	CD31+,CD34+,PCK-,EMA-,Ki-67-5%	*TFE3-*rearranged(FISH); *YAP1::TFE3*(PCR)	biopsy chemotherapy	centrum metastasis at diagnosis; AWD/53mo
5	33/M	multiple intrahepatic lesions	abdominal pain	9	cords and nests of epithelioid and spindle cells with intracytoplasmic vacuoles in a myxohyaline stroma	–	–	CD31+,CD34+,PCK-,EMA-,Ki-67-5%	*WWTR1::CAMTA1*(FISH)	biopsy chemotherapy	AWD/32mo
6	38/M	right liver	asymptomatic	2	cords and nests of epithelioid cells, signet ring-like lumen with erythrocytes	–	–	CD31+,CD34+,PCK-,EMA-,Ki-67-5%	*WWTR1::CAMTA1*(FISH)	SR	NED/39mo
7	44/F	multiple intrahepatic lesions	asymptomatic	3.3	medium sized epithelioid cells, eosinophilic cytoplasm with vascular lumens containing erythrocytes	–	–	CD31+,CD34+,PCK-,Ki-67-5%	*WWTR1::CAMTA1*(FISH&NGS)	SR chemotherapy	NED/54mo

F, female; M, male; FISH, fluorescence *in situ* hybridization; PCR, polymerase chain reaction; NGS, next-generation sequencing; AWD, alive with disease; mo, month; NED, no evidence of disease; NA, not available; SR, surgical resection; -, negative; +, positive.

### Immunohistochemical findings

Immunohistochemically, nuclear CAMTA1 was negative in all the 7 EHE tumors, but the positive control showed positivity. Among the 7 cases, 4 cases exhibited TFE3 positivity and 3 cases were also TFE3-immunonegative. In the 4 TFE3-positive cases, 2 cases showed diffuse nuclear staining (3+) with moderate to strong intensity for TFE3 (case 1 and 2), another 2 cases showed focal nuclear staining (2+) with moderate to strong (case 4) or moderate intensity (case 3). The vascular markers CD31, ERG, and CD34 were generally and strongly expressed in all the 7 cases, and the epithelial markers EMA, cytokeratin was negative in all tested cases. The Ki-67 index was about 5% of the neoplastic cells in 6 cases but was up to 15% in case1.

### Molecular results

In the 4 CAMTA1-immunonegative but TFE3-immunopositve cases, all cases were positive for *TFE3* rearrangement by FISH, and further demonstrated by RT-PCR. In one case, *YAP1::TFE3* fusion was identified by NGS (case 3). All the 4 cases harbored gene fusions between *YAP1* exon 1 (NM_001130145.2) and *TFE3* exon 4(NM_006521.4).

Among the 3 cases who were immunonegative for both CAMTA1 and TFE3, 3 cases were subjected to FISH analysis, and *WWTR1::CAMTA1* fusion gene were identified in each of the 3 cases. In case 7, *WWTR1::CAMTA1* fusion was also identified by NGS at both the DNA and RNA levels with the fusion between *WWTR1* exon 2 and *CAMTA1* exon 9 (*WWTR1*:NM_015472; *CAMTA1*: NM_015215). Moreover, the NGS identified a novel breakpoint at nucleotide positions 1663 of *CAMTA1* gene.

### Histopathological characteristics

#### *TFE3* rearranged EHE

**Figure 1:**
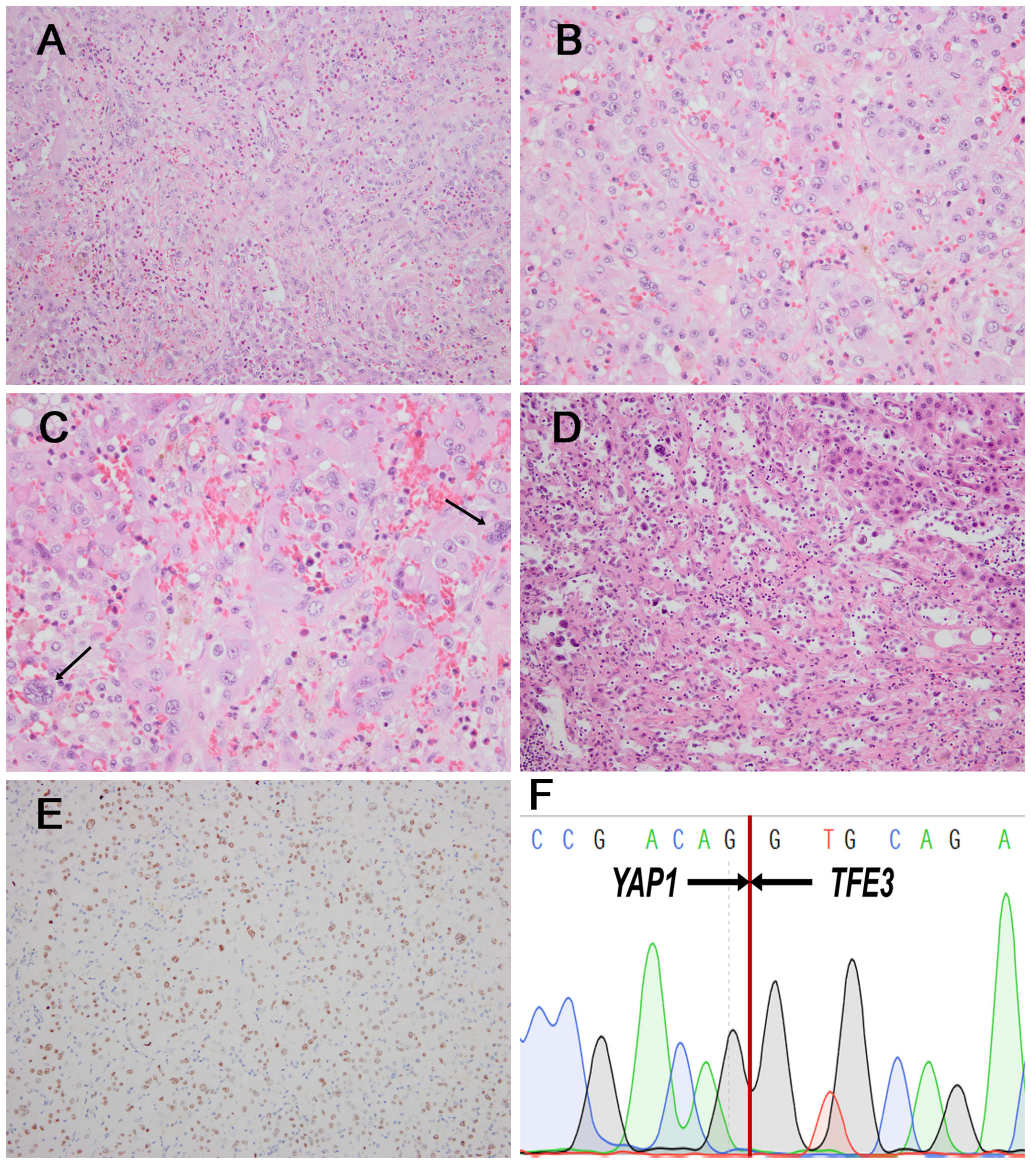
Morphological features of *TFE3* rearranged EHE case and corresponding immunohistochemical and genetic results. **(A)** Nest or sheets of epithelioid cells against the background of inflammatory cell (H&E, ×200). **(B)** The tumor cells had abundant, eosinophilic to vacuolated cytoplasm with prominent nucleoli (H&E, ×400). **(C)** A few cells with nuclear pleomorphism(arrow) were seen around (H&E, ×400). **(D)** Some areas formed pseudoalveolar architecture (H&E, ×200). **(E)** TFE3 positivity was identified in the case (×200), and Sanger sequencing demonstrated the presence of the *YAP1::TFE3* fusion gene **(F)**.

**Figure 2:**
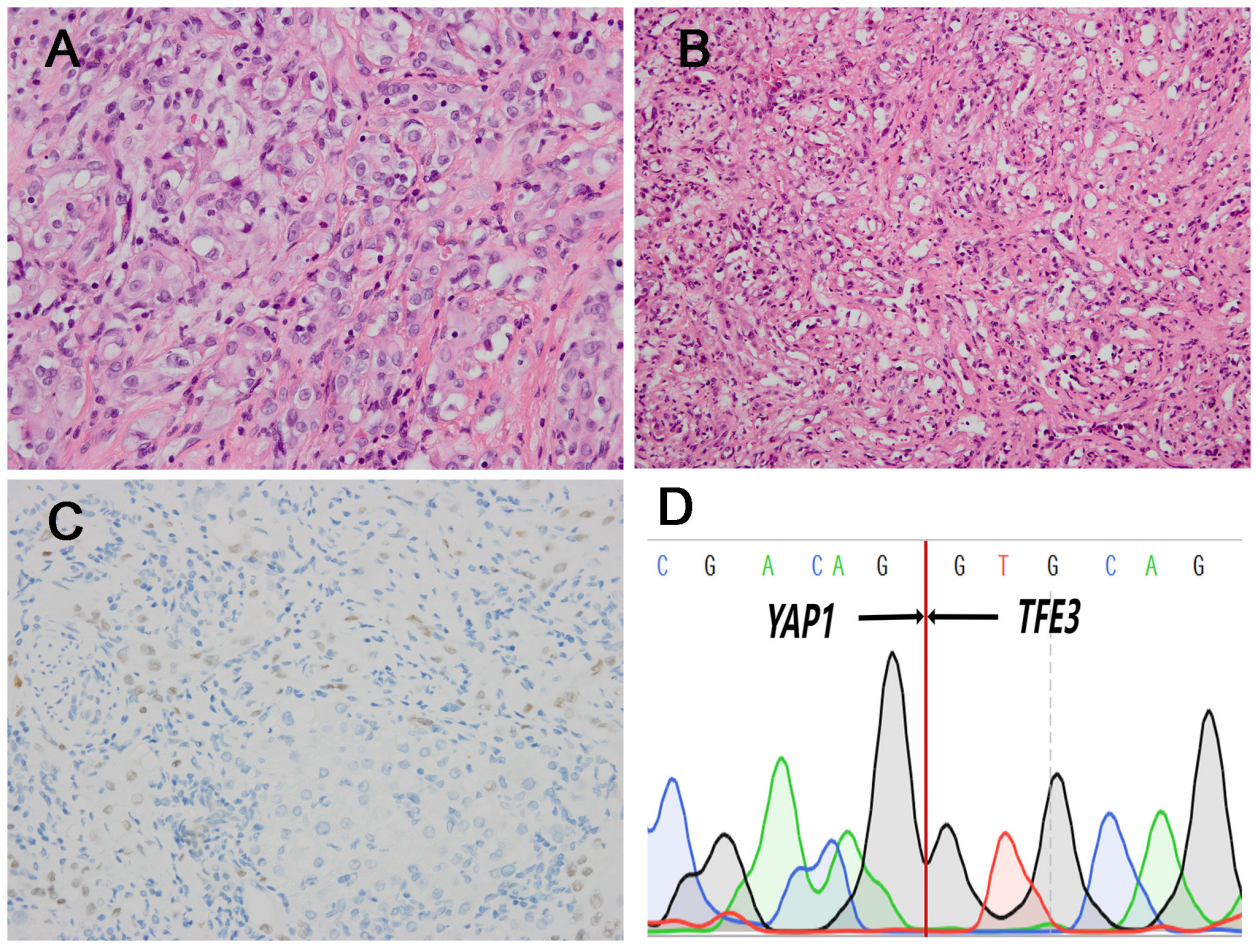
Histopathological features of one *TFE3* rearranged (case3) which had lower atypia and corresponding immunohistochemical and genetic results. **(A)** Medium-sized epithelioid cells exhibited slightly rich cytoplasm, round nuclei and conspicuous nucleoli (H&E, ×400). **(B)** There were the formation of vascular channels containing erythrocytes (H&E, ×200). **(C)** The case was positive for TFE3 IHC (×200). **(D)** Sanger sequencing confirmed the presence of *YAP1::TFE3* fusion gene.

In the 4 *TFE3*-rearranged EHE cases, two cases (case 1 and 2) were composed of solid sheets and nests of large epithelioid cells in an inflammatory background (**Figure 1A**). The tumor cells had prominent nucleoli, with abundant, eosinophilic to vacuolated cytoplasm (**Figure 1B**), and nuclear pleomorphism can be seen (**Figure 1C**). The dominant feature was the well-formed, numerous vascular channels, and some areas formed pseudoalveolar-like architecture (**Figure 1D**). Both of the cases exhibited TFE3 immunopositivity and *YAP1*::*TFE3* fusion gene (**Figures 1E–F**). In case 3, the tumor intermingled with liver parenchyma in a solid growth pattern with medium-sized epithelioid cells, exhibiting slightly rich cytoplasm, round nuclei and conspicuous nucleoli (**Figure 2A**). Mature vessel lumen formation which contained prominent erythrocytes, could be identified in some area (**Figure 2B**). This case harbored focal nuclear staining (2+) for TFE3 and *YAP1::TFE3* fusion gene (**Figures 2C, D**).

**Figure 3:**
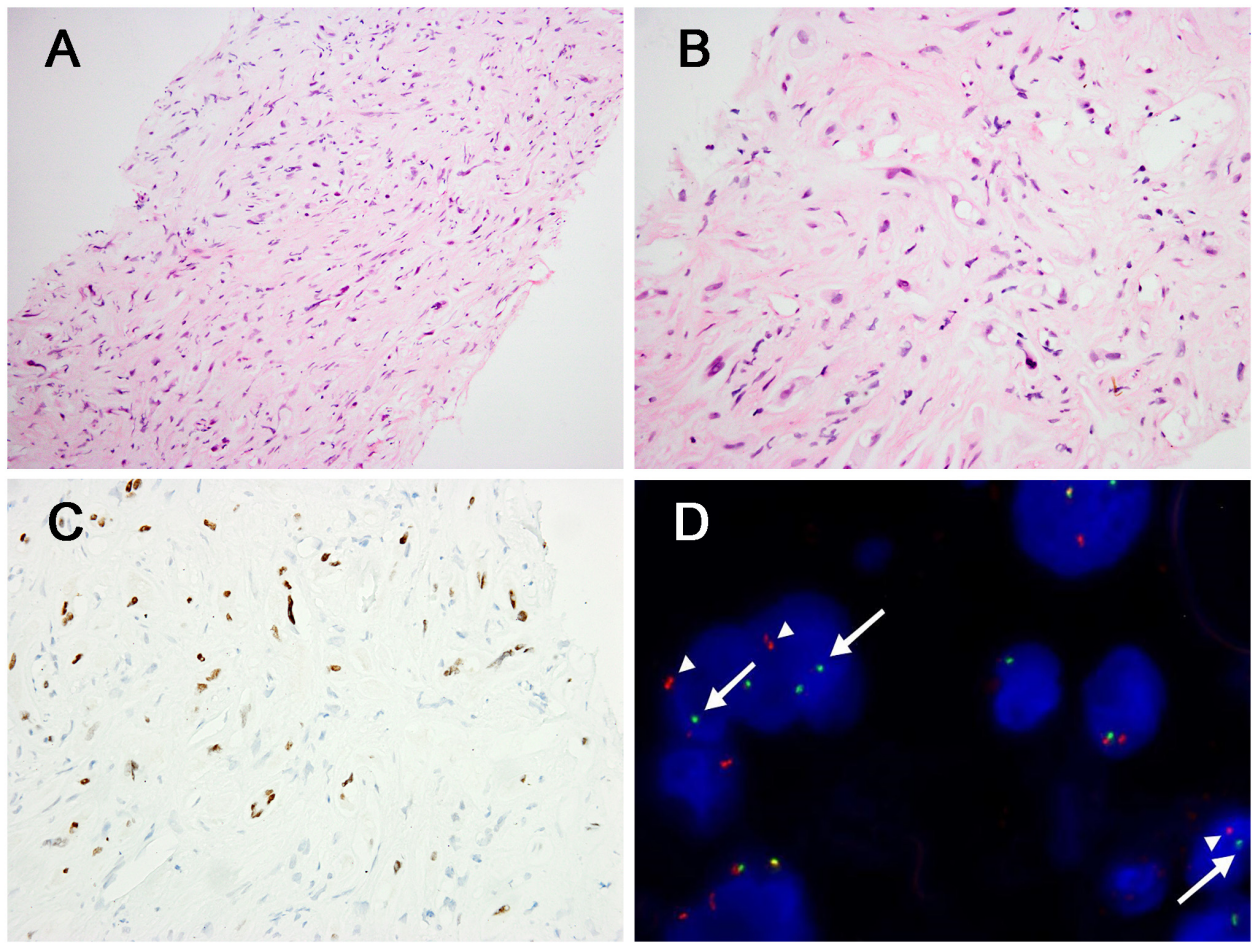
Morphological features of *TFE3* rearranged (case4) and corresponding immunohistochemical and genetic results. **(A)** The tumor cells arranged in discohesive strands or single cells in the collagen matrix, with indistinct cell borders (H&E, ×200). **(B)** Some tumors cells had intracytoplasmic vacuoles and the presence of signet-ring lumen (H&E, ×400). **(C)** The case showed positivity for TFE3 (×400). **(D)** FISH demonstrated the presence of *TFE3* gene rearrangement in the neoplastic cells [separation of the red (white arrowhead) and green (white arrow) signals].

In case 4, the tumor cells arranged in discohesive strands or single cells in the collagen matrix. The lesion comprised spindle cells with mild atypia, fusiform nuclei, inconspicuous nucleoli, and indistinct cell borders (**Figure 3A**). In some cells, intracytoplasmic vacuoles were present with lumina formation, exhibiting signet-ring like appearance (**Figure 3B**). This case also showed nuclear staining (2+) for TFE3 and *TFE3*-rearrangement (**Figures 3C, D**). The mitotic figures ranged from 0 to 2 per 10 high-power fields (HPFs) in the 4 cases. Significant necrosis was not identified in four cases.

#### *CAMTA1* rearranged EHE

**Figure 4:**
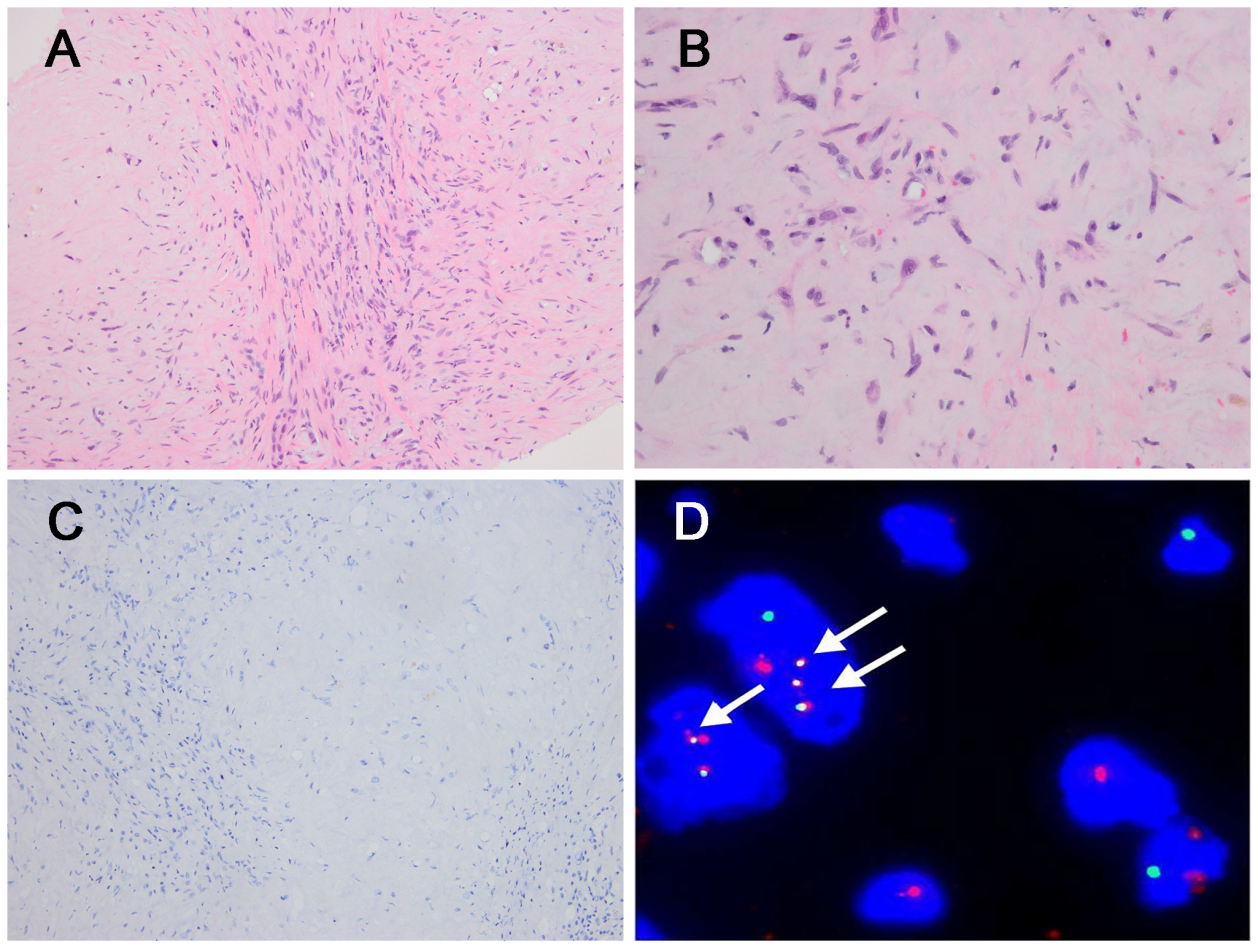
Morphological features of *CAMTA1* rearranged and immunohistochemical and genetic results. **(A)** In some areas, strands or single epithelioid and spindled cells in a myxohyaline stroma (H&E, ×200). **(B)** Tumor cells had fusiform and oval nuclei with mild atypia, and intracytoplasmic vacuoles, imparting a signet-ring like appearance (H&E, ×400). **(C)** The tumor was immunonegative for CAMTA1 (×200). **(D)** FISH revealed *WWTR1::CAMTA1* fusion (arrow).

**Figure 5:**
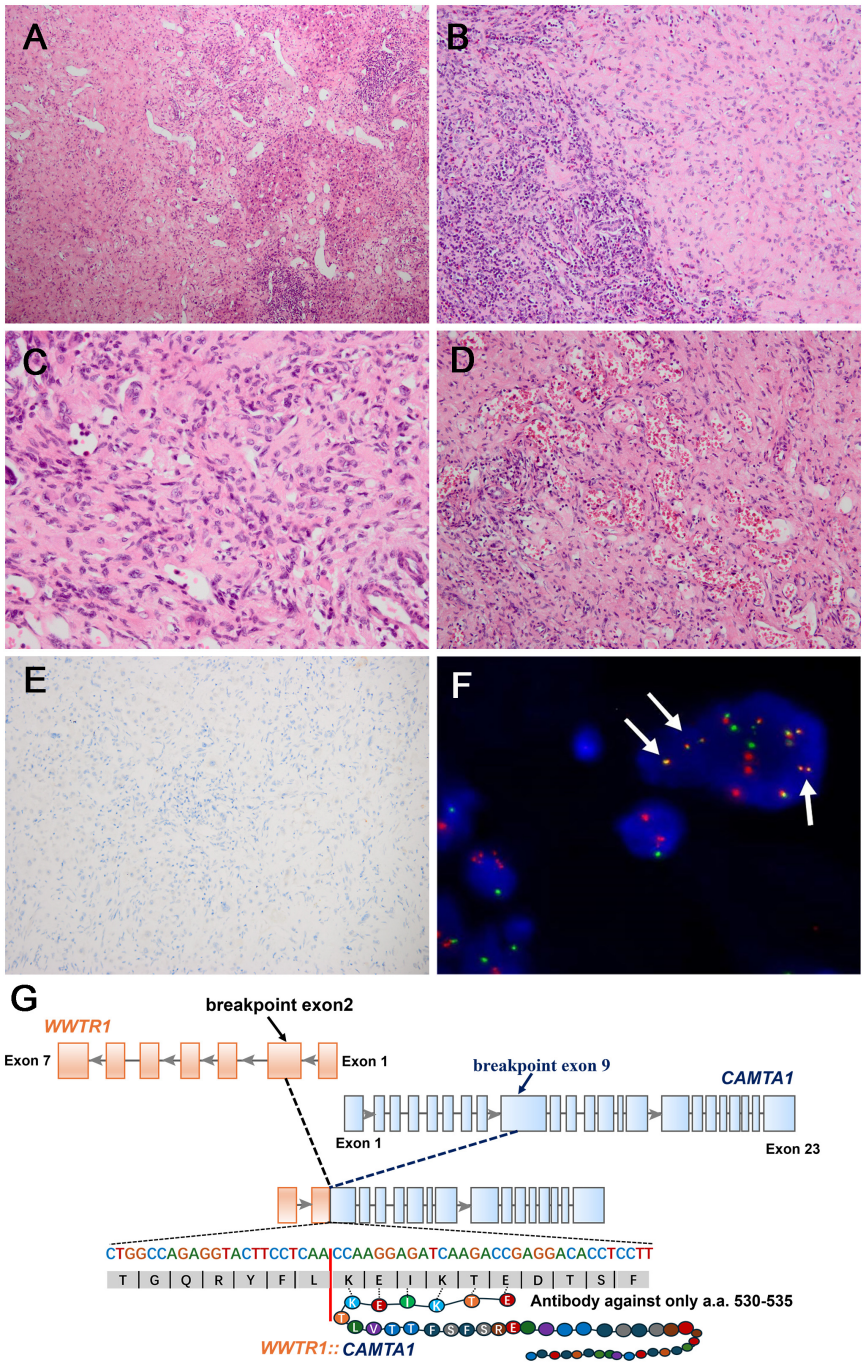
Morphological features of one *CAMTA1* rearranged (case7) and immunohistochemical and genetic results. **(A)** At low magnification, infiltrative growth into liver parenchyma can be seen (H&E, ×100). **(B)** The tumor showed solid growth in a sclerotic matrix with lymphocyte infiltration (H&E, ×200). **(C)** Tumor cells had round nuclei with moderate atypia and conspicuous nucleoli, with moderate amounts of eosinophilic cytoplasm (H&E, ×400). **(D)** Vascular channels containing red blood cells can be seen in some areas (H&E, ×200). **(E)** Tumor cells were immunonegative for CAMTA1 (×200). **(F)** FISH revealed *WWTR1::CAMTA1* fusion, (arrow). **(G)** Schematic diagram of the *WWTR1::CAMTA1* fusion identified by NGS. The breakpoints were in the *WWTR1* (exon2) and *CAMTA1*(exon9) and the fusion transcript only had 6 amino acids that could be recognized by the CAMTA1 antibody.

In the three *CAMTA1* rearranged cases, case 5 and case 6 showed discohesive strands or single epithelioid or spindled cells in a myxohyaline stroma (**Figure 4A**). Tumor cells had fusiform and oval nuclei with mild atypia, and intracytoplasmic vacuoles, imparting a signet-ring like appearance (**Figure 4B**), with CAMTA1-immunonegativity but *WWTR1::CAMTA1* fusion (**Figures 4C, D**). Another *CAMTA1* rearranged case (case 7) had higher atypia and the tumor exhibited infiltrative growth into liver parenchyma (**Figure 5A**). The tumor showed solid growth pattern in a sclerotic matrix with lymphocyte infiltration (**Figure 5B**). Medium-sized epithelioid cells had round nuclei with moderate atypia and conspicuous nucleoli, with moderate amounts of eosinophilic cytoplasm (**Figure 5C**). In some area, the tumor showed vascular lumens that contain erythrocytes (**Figure 5D**). Nuclear expression of CAMTA1 was negative while *WWTR1::CAMTA1* fusion was identified by FISH and NGS (**Figures 5E–G**). Nuclear pleomorphism can be seen in case 7 and necrosis can be seen in case 5, but significant mitotic activity was not identified in three cases.

#### Treatment and prognosis

Among 7 cases, 5 cases underwent surgical resection, one of which was followed by adjuvant chemotherapy. Two cases were biopsied and treated with chemotherapy. (case 4 with Epirubicin and case 5 with Doxorubicin and Sirolimus). Follow-up information was available for 6 patients (6/7, 85.7%) with a median follow-up duration of 46 months (range 16-86 months). Among the 6 patients, 1 patient had centrum metastases at initial diagnosis. Four patients (4/6, 66.7%) were alive with no evidence of disease, and 2 patients (2/6, 33.3%) were alive with disease.

## Discussion

EHE is a rare malignant vascular neoplasm which was first described by Weiss and Enzinger in 1982 (13). The majority of EHEs (>90%) are characterized by *WWTR1::CAMTA1* fusions, therefore, CAMTA1 IHC is commonly utilized as a diagnostic tool for EHE. Subsequently, a small subset EHE cases were found to harbor *YAP1*::*TFE3* fusion gene and negative for CAMTA1 IHC but showed positivity to TFE3. Most CAMTA1-immunonegative cases are composed of the *TFE3* subtype, however, in our clinical practice, CAMTA1-immunonegative but *CAMTA1* gene rearrangements cases were identified, and further literature review found more such cases (8, 9). Moreover, with the development of NGS, a few EHE cases were found to be negative for *CAMTA1* rearrangement, but with variant *WWTR1* fusion, including *WWTR1::MAML2* (n=2), *WWTR1::ACTL6A* (n=2), *WWTR1::TFE3* (n=1) (**Table 2**) (8, 10, 11). Furthermore, there existed EHE cases with *WWTR1* rearrangement, but NGS failed to identify fusion gene candidate (11). So far, no studies have systematically reported these peculiar cases. Hence, we focused on liver, one of the most common sites of EHE, to further explore the clinicopathological and molecular features of these CAMTA1-immunonegative tumors.

**Table 2:** Clinicopathological and molecular features of historical EHE cases with CAMTA1-immunonegativity or variant *WWTR1* gene rearrangement.

Case no	Reference	Age/Sex	Site	Size(cm)	Morphology	Immunohistochemistry	Molecular results	Treatment	Outcome
1	Shibayama et al. (8)	NA	NA	NA	NA	CAMTA1-	*WWTR1::CAMTA1*(FISH)	NA	NA
2		NA	NA	NA	NA	CAMTA1-	*WWTR1::CAMTA1*(FISH)	NA	NA
3		58/F	heart	4.4	EHE with atypical histology: high nuclear atypia and increased mitotic activity	CAMTA1-	*WWTR1::ACTL6A*(FISH/RNA seq)	debulking surgery	DOD/7mo
4	Yang et al. (9)	49/M	humerus	NA	NA	CD31+,CD34+,ERG+,CAMTA1-	*WWTR1::CAMTA1*(FISH)	NA	AWD/17mo
5		49/F	liver	NA	NA	CD31+,CD34+,ERG+,CAMTA1-	*WWTR1::CAMTA1*(FISH)	NA	NED/10mo
6	Li et al. (10)	26/F	lung	1.8	classic EHE areas, areas resembling *TFE3*-fused EHE and tumor cells with prominent vacuolated cytoplasm admixed with extravasated erythrocytes	CD31+,ERG+,CAMTA1-	*WWTR1::TFE3* (RNA seq)	radiotherapy	brain metastasis at 7mo; AWD/24mo
7	Suurmeijer et al. (11)	76/F	heart	NA	classic EHE histologic features	CD31+,ERG+	*WWTR1::MAML2*(FISH/RNA seq)	NA	NA
8		21/M	bone	NA	classic EHE histologic features	CD31+,ERG+	*WWTR1::MAML2*(FISH)	resection	NED/70mo
9		73/F	heart	NA	EHE with malignant features: large epithelioid cells with significant nuclear atypia, brisk mitotic activity	CD31+,ERG+	*WWTR1::ACTL6A*(FISH/RNA seq)	NA	DOD/9mo
10		72/F	heart	NA	classic EHE histologic features	CD31+,ERG+	*WWTR1-*rearrangement(FISH )	chemotherapy	soft tissue metastases; DOD/15mo
11		67/M	heart	NA	classic EHE histologic features	CD31+,ERG+	*WWTR1-*rearrangement(FISH )	NA	lung metastases at diagnosis
12		65/M	pelvic	NA	EHE with malignant features: nuclear pleomorphism and easily discerned mitoses	CD31+,ERG+	*WWTR1-* rearrangement(FISH)	NA	recent case

F, female; M, male; FISH, fluorescence *in situ* hybridization; RNA-seq, RNA sequencing; mo, month; AWD, alive with disease; DOD, died of disease; NED, no evidence of disease; NA, not available; -, negative; +, positive.

In our series, 4 CATMA1-immunonegative hepatic cases were proved to be *TFE3*-subtype EHE, including 2 males and 2 females with a median age of 60 years. There were 17 *TFE3*-subtype hepatic EHE cases that had been reported in English literatures (**Table 3**) (14–23). Sixteen (including our study) historical, hepatic *TFE3*-subtype EHEs had clinical information, including 8 males and 8 females. Histologically, 2 *TFE3*-rearranged cases (case1 and 2) in our cohort displayed classic *TFE3*-subtype morphologic change, and case 3 also showed *TFE3*-subtype morphology with a certain degree variation, harboring a lower degree of atypia, smaller tumor cells and moderate cytoplasm. Furthermore, case 4 only displayed mild atypia that could potentially be misdiagnosed as *CAMTA1*-subtype EHE morphologically, harboring cells with intracytoplasmic vacuoles and lumina formation, presenting a signet-ring like appearance. In the hepatic *TFE3*-subtype cases we reviewed, morphological manifestations were available in 7 cases, of which 4 showed classical *TFE3*-type morphology and only 1 case had similar morphology to case 4, with morphologic change as *CAMTA1* subtype EHE (23). However, unlike our case, this case was not genetically confirmed. Besides, the morphology of the other 2 historical *TFE3*-subtype cases were uncommon, with dual growth pattern. One case exhibited pseudo-alveolar architecture and cords, nests, and single neoplastic cells in myxoid area simultaneously (14). Another case had classic *TFE3*-subytpe area and focal area resembling nodular hyperplasia (17). Both in our series and historical cases, the hepatic *TFE3*-subtype EHE harbored unusual morphologic change, and great care with ancillary analysis should be taken to avoid misdiagnosis.

**Table 3:** Clinicopathological and molecular features of hepatic *YAP1::TFE3*-fused EHEs in the published literature.

Case no	Reference	Age/Sex	Location	Size (cm)	Morphology	Immunohistochemistry		Gene		Treatment	Outcome
						CAMTA1	TFE3	*CAMTA1*	*TFE3*		
1	Kuo et al. (14)	39/F	liver	4	dual growth pattern: pseudoalveolar architecture and discohesive cords and single tumor cells	NA	+	*NA*	*TFE3-*rearranged (FISH)	LT	NED/156mo
2	Thway et al. (15)	40/F	liver, lung, soft tissue	NA	NA of liver lesion	+	–	Non-rearranged	*TFE3*-rearranged (FISH)	SR/chemotherapy	AWD/78 mo
3	Jung et al. (16)	37/M	liver	7.5	NA	–	+	NA	NA	SR	NED/48mo
4	Lotfalla et al. (17)	65/F	liver	3	dual histologic pattern; vascular channels, discohesive cords, and small irregular central hyalinized scars	–	+	Non-rearranged	*YAP1::TFE3(*FISH,RNA sequencing)	LT	NA
5	Rosenbaum et al. (18)	NA	liver, lung	NA	NA	NA	+	NA	*YAP1::TFE3*(FISH)	NA	NA
6		NA	liver, lung	NA	NA	NA	+	NA	*YAP1::TFE3*(FISH)	NA	NA
7		NA	liver, lung, bone	NA	NA	NA	+	NA	*YAP1::TFE3*(FISH)	NA	NA
8		NA	liver, lung, bone	NA	NA	NA	+	NA	*YAP1::TFE3*(FISH)	NA	NA
9		NA	liver, lung, soft tissue	NA	NA	NA	+	NA	*YAP1::TFE3*(FISH)	NA	NA
10	Xu et al. (19)	35/M	liver	NA	NA	NA	+	NA	NA	LH	NED/60mo
11		67/F	liver	NA	NA	NA	+	NA	NA	LH	NED/48mo
12		55/M	liver	5.8	NA	NA	+	NA	NA	LH/chemotherapy	NED/9mo
13	Bourgeau et al. (20)	18/M	liver, lung	NA	classical *TFE3*-type: tumor cells in solid growth pattern with moderate eosinophilic cytoplasm and enlarged irregular nuclei	NA	+	NA	*YAP1::TFE3*(targeted sequencing panel)	chemotherapy	AWD/9mo
14	Dermawan et al. (21)	37/M	liver	4	classical *TFE3*-type: solid sheets of epithelioid cells with abundant cytoplasm	NA	NA	NA	*YAP1::TFE3*(FISH)	SR/chemotherapy/RA	DOD/27mo
15		36/F	liver	5.6	classical *TFE3*-type: solid sheets of coalescing nests, large epithelioid cells with abundant cytoplasm	NA	NA	NA	*YAP1::TFE3*(PCR)	chemotherapy/RA	Bone metastasis at presentation; AWD/21mo
16	Shishimoto et al. (22)	71/M	liver	3.5	classical *TFE3*-type: round and spindle shaped epithelial atypical cells in a sarcomatoid fashion	NA	+	NA	NA	biliary drainage	AWD/28mo
17	Ribeiro et al. (23)	17/F	liver, lung	12.9	intracytoplasmic vacuoles containing red blood cells with fibrosis and hyalinization	NA	+	NA	NA	chemotherapy/RA	NA

F, female; M, male; FISH, fluorescence *in situ* hybridization; PCR, polymerase chain reaction; SR, surgical resection; LH, laparoscopic hepatectomy; LT, liver transplantation; RA, radiation therapy; NA, not available; mo, month; AWD, alive with disease; NED, no evidence of disease; DOD, dead of disease; -, negative; +, positive.

Immunohistochemically, nuclear TFE3 was uniformly expressed, while CAMTA1 was negative in the 4 cases. In terms of genetics, all 4 cases harbored *YAP1::TFE3* fusion and had gene fusions between *YAP1* exon 1 and *TFE3* exon 4. Of the 17 historical cases, 11 had genetic information, harboring *YAP1::TFE3* fusions gene or *TFE3* rearrangement. Three of the 11 cases had exon information, involving *YAP1* exon 1 and *TFE3* exon 4, like our report. The above data was also similar to the previous largest *TFE3*-subtype EHEs study, among which the majority (14/16, 88%) cases had *YAP1* exon 1 fused to *TFE3* exon 4 (21). These results suggested the TFE3 immunohistochemistry could aid in the EHE diagnosis and further genetic analysis could help to establish the final diagnosis.

In our study, another 3 hepatic CAMTA1-immunonegative cases were proved to be positive for *WWTR1::CAMTA1* gene fusion. Four historical cases harbored similar immunohistochemical and genetic change, with *CAMTA1* rearrangement and negative CAMTA1 immunoreactivity (8, 9). However, the 4 cases lacked the description of their morphology. In our study, 2 of the 3 cases exhibited classic histology of *CAMTA1*-subtype, showing nests of epithelioid tumor cells with small, oval nuclei and intracytoplasmic vacuoles. It needs to be pointed out, another 1 case (case 7), unlike characteristic EHE, showing the morphology analogous to *TFE3*-subtype, with medium-sized epithelioid cells having moderate amounts of eosinophilic cytoplasm and vascular lumen, containing erythrocytes. More cases and further studies and needed to clarify the pathological feature of these peculiar cases. More importantly, when meeting such peculiar and challenging cases with unusual morphologic and immunohistochemical results, molecular studies such as FISH and furthermore, NGS are needed to establish the diagnosis and reveal the reason for these changes.

In this study, we identified a novel fusion between *WWTR1* exon 2 and *CAMTA1* exon 9 in one case (case 7) through NGS. This breakpoint is located at the 1663 nucleotide of *CAMTA1* gene (NM_015215.4), a previously unreported site, and the breakpoint of *WWTR1* gene is identical to previous studies. The current CAMTA1 antibody only recognizes 84 amino acids encoded by nucleotides 1428 to 1682 of the *CAMTA1* gene, which is located downstream of all previously reported *CAMTA1* breakpoints (4, 6, 24–27). However, the antibody recognition sequence is located upstream of the breakpoint of case 7, with only 20bp nucleotide overlapped and corresponding to 6 amino acids (**Figure 5G**). This result suggested that the uncommon fusion point might result in the negative IHC result. This finding underscores the importance of understanding the precise location of fusion points, as breakpoint could impact the gene translation and antibody recognition and may lead to unexpected results. Additionally, the CAMTA1 immuno-negativity may also occurred in *TFE3*-rearranged EHE cases and more rarely, in cases with variant *WWTR1* gene rearrangements. Hence, when meeting such challenging cases with uncommon IHC results, the FISH and NGS is valuable to assist the establishment of correct diagnosis.

The histopathologic morphology of hepatic EHE is diverse and heterogeneous, particularly in small biopsy samples, and it is usually too difficult to confirm endothelial differentiation and to exclude histologic mimics. EHE includes a wide range of differential diagnoses, including poorly differentiated adenocarcinoma, perivascular epithelioid cell tumor (PEComa), epithelioid angiosarcoma and so on.

Poorly differentiated adenocarcinoma can also have distinct signet-ring cell components and morphologically overlapped with EHE. The typical features of adenocarcinoma are a large number of cells with intracytoplasmic vacuoles containing mucus, readily confirmed by epithelial mucinous histochemical staining such as PAS (28). Immunohistochemically, adenocarcinoma expresses epithelial markers such as CK and EMA, but negative for CD31, CD34 and especially CAMTA1 and TFE3. Genetically, *CAMTA1* or *TFE3*-rearrangement is present in EHE, but not in poorly differentiated adenocarcinomas.

PEComa also has spindle or epithelioid cells arranged in fascicular or nested distribution, like EHE, but the former lacks evident vascular differentiation. PEComa exhibit expression of melanocyte markers such as HMB45 and Melan-A, as well as myogenic markers including SMA and desmin, while they do not express the endothelial marker CD34 (29). Generally, specific immunohistochemical markers can be utilized for distinguishing between these two entities. Since both have *TFE3* gene break, they can be identified by gene sequencing if necessary to prevent misdiagnosis or missed diagnosis. Unlike *YAP1::TFE3* EHEs, PEComa is frequently accompanied by *SFPQ::TFE3* fusion.

The distinction between EHE and epithelioid angiosarcoma can be challenging due to their similar histological change with large epithelioid cells, eosinophilic cytoplasm, distinct nucleoli, and occasional intracytoplasmic vacuoles containing erythrocytes. However, the latter exhibits higher atypia and marked mitosis. Furthermore, CAMTA1 or TFE3 immunohistochemistry may provide valuable insights. Moreover, confirming the presence of *WWTR1::CAMTA1* or *YAP1::TFE3* gene fusion in the tumor would offer more convincing evidence.

**Figure 6:**
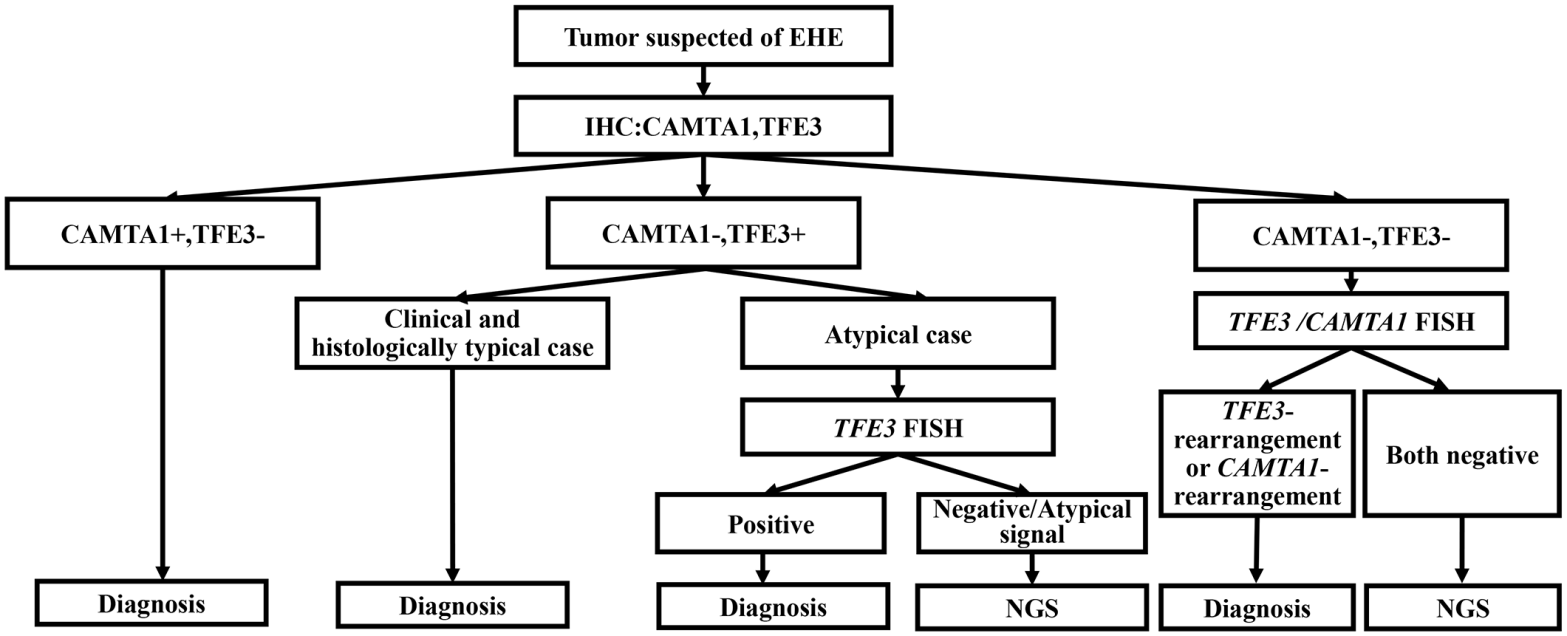
Diagnostic algorithm for soft tissue tumors suspected of EHE.

In clinical practice, the diagnosis of EHE is mainly based on the histological evaluation and IHC results, and for the challenging cases with ambiguous morphology or unexpected IHC profile, the FISH for *CAMTA1* and *TFE3* should be performed, and furthermore, NGS is recommended for cases which is highly suspected for EHE to make the final diagnosis. We recommend the diagnostic algorithm shown in the figure to minimize misdiagnosis (**Figure 6**).

Owing to the rarity and heterologous clinical course of hepatic EHE, there is no well-established treatment strategy for such entity. In our series, 4 patients were treated with surgical resection only, 1 patient received surgical resection and chemotherapy and 2 patients underwent chemotherapy. According to previous large series studies of hepatic EHE, the common treatment modalities include hepatic resection, liver transplantation, ablations, chemotherapy, and radiotherapy. Hepatic resection is recommended for resectable intrahepatic lesions, and liver transplantation is considered to be the first choice for the treatment of tumor patients when there is intrahepatic metastasis or the tumor is too large to be resected (30, 31). In addition, adjuvant chemoradiotherapy is recommended when metastasis occurs.

In cases of EHE, the discovery of the *WWTR1::CAMTA1* and *YAP1::TFE3* fusion genes has provided a new perspective for the treatment of EHE. *WWTR1* and *YAP1* are key components of the Hippo signaling pathway, playing a central role in a variety of physiological and pathological processes. The *WWTR1::CAMTA1* fusion gene can induce endothelial cells to transform into vascular tumors with EHE characteristics. This process involves *WWTR1* (TAZ)*::CAMTA1* as a continuously activated form of TAZ, which is mainly located in the cell nucleus and activates its pro-tumor transcription program. Similarly, the carcinogenic potential of the *YAP1::TFE3* fusion gene also requires interaction with TEAD. The fusion protein utilizes the transcriptional activation domain and nuclear localization sequence of TFE3, binding to DNA through the TEAD binding site of YAP1, forming a continuously activated chimeric transcription factor (32). Additionally, trametinib, an MEK inhibitor, has demonstrated certain therapeutic effects in EHE patients with the *WWTR1::CAMTA1* fusion (33). In the future, it is necessary to further collect and analyze cases to gain a deeper understanding of the molecular basis of EHE, in order to provide more precise treatment strategies for patients.

In our series, 4 patients (4/6; 66.7%) were alive with no evidence of disease, and 2 patients (2/6;33.3%) were alive with disease. Previous studies found the 5-year overall survival rate of hepatic EHE ranging from 57% to 67% (18, 34). Pathologic parameters including tumor size >3 cm, mitotic activity >3 mitoses per 50 high-powered fields and histologic grade were associated with worse outcome of EHE patients (18, 35). Moreover, a recent large series study discovered the *CAMTA1*-subtype EHE correlated with a worse prognosis compared to *TFE3* subtype, with the 5-year overall survival (OS) as 59% versus 86%, respectively (18). Hence, we carefully reviewed the historical hepatic EHE cases and found 74 *CAMTA1* subtype and 13 *TFE3* subtype EHE cases with available follow-up information (including our cases)) (9, 16, 26, 31, 36–45). The survival results showed 10 of 74 (13.5%) *CAMTA1* subtype cases died of disease and only 1 of 13 (7.7%) *TFE3*-subtype EHEs succumbed to disease. Combined with this study and retrospective data analysis, most patients with hepatic EHE have a good prognosis, and *TFE3* subtype cases seems to harbor a favorable outcome. While the prognosis difference between the *TFE3*-subtype and *CAMTA1*-subtype hepatic EHE still needs to be further explored in a larger study cohort.

In summary, our study reported a series of 7 peculiar, CAMTA1-immunonegative hepatic EHE cases and most of them were *TFE3*-rearranged EHEs. Moreover, our study showed that there existed the CAMTA1-immunonegative but *CAMTA1*-rearranged EHE cases. Therefore, the diagnosis of EHE should be based on histological morphology, combined with the dual detection of CAMTA1 and TFE3 IHC, and if necessary, supplemented by comprehensive analysis, including FISH and NGS for the genetic aberration, to ensure correct diagnosis.

## Data Availability

The data presented in the study are deposited in the NCBI database, accession number PV083432.
